# Drug breakthrough offers hope to arthritis sufferers: qualitative analysis of medical research in UK newspapers

**DOI:** 10.1111/hex.12460

**Published:** 2016-05-04

**Authors:** Helen Hanson, Nicola O'Brien, Paul Whybrow, John D Isaacs, Tim Rapley

**Affiliations:** ^1^ National Institute for Health Research Newcastle Biomedical Research CentreNewcastle upon Tyne Hospitals NHS Foundation Trust and Newcastle UniversityNewcastle upon TyneUK; ^2^ Institute of Health and SocietyNewcastle UniversityNewcastle upon TyneUK; ^3^ School of Social and Community MedicineUniversity of BristolBristolUK

**Keywords:** arthritis, health research, mass media, newspaper, patient narrative, research participation

## Abstract

**Background:**

Newspaper stories can impact behaviours, particularly in relation to research participation. It is therefore important to understand the narratives presented and ways in which these are received. Some work to date assumes journalism transmits existing medical knowledge to a passive audience. This study aimed to explore how newspaper articles present stories about medical research and how people interpret and use them.

**Design:**

Qualitative research methods were employed to analyse two data sets: newspaper articles relating to ‘rheumatoid arthritis’ and ‘research’ from UK local and national news sources; and existing transcripts of interviews with patients with rheumatoid arthritis and their carers.

**Results:**

Newspapers present a positive account of medical research, through a simple narrative with three essential components: an ‘innovation’ offers ‘hope’ in the context of ‘burden’. Patients frequently feature as passive subjects without attributed opinions. Few articles include patients’ experiences of research involvement. Patients with rheumatoid arthritis and their carers read articles about medical research critically, often with cynicism and drawing on other sources for interpretation.

**Conclusions:**

An understanding of the simple, positive narrative of medical research found in newspaper articles may enable researchers to gain mass media exposure for their work and challenge this typical style of reporting. The critical and cynical ways patients and carers read stories about medical research suggest that concerns about newspaper articles misinforming the public may be overstated, but any effect on research engagement is unknown. Newspaper articles rarely present patients’ views or their experiences of research, and this can be conceptualized as ‘depersonalization bias’.

## Background

In the UK, 87% of adults read newspapers, either in traditional paper or electronic format.^1^ People use newspapers as a source of information about health issues including new medical research.^2^ While media research has long illustrated the impact of newspapers on public knowledge,^3^ there are recent concerns about the media's role in shaping the public understanding of health and medicine.^4^ Newspaper stories have the potential to dramatically impact the behaviours of the reader, particularly in relation to stimulating medical research interest and participation enquiries, as demonstrated in the following examples. In March 2006, six men had severe and life‐threatening reactions during a clinical trial of the compound TGN 1412, a treatment being developed to target leukaemia or rheumatoid arthritis (RA). They were in a private research unit at Northwick Park Hospital in North West London. Following these events, which generated huge media attention, there was an unexpected increase in general enquiries about participation to several charities and commercial research organizations.^5^ A doctor who gave an interview to a leading Dutch newspaper about his trial of a new surgical implant, reported being inundated with 1000 enquiries from people wanting to take part in his trial.^6^ Researchers have a duty to publish and publicize their work. To do this responsibly and effectively, they should be aware of how newspapers tend to present stories about medical research and the potential impact of these stories on patients and carers.

To assess the quality and accuracy of newspaper health stories, Robinson *et al*.^7^ reviewed eight UK newspapers, finding a high degree of variability and raising concerns about medical journalism. Articles commonly failed to include information about risks and costs associated with treatments. Similar results have been reported in Europe,^8^ the USA^9^, ^10^ and Canada.^11^ Aggarwal *et al*.^12^ explored the views of European journalists reporting cancer research and described a ‘schism’, with limited communication and understanding between the cancer research and media communities. These studies take a linear reflection perspective, based on the assumption that medical knowledge is created by experts and transmitted to the uninformed public by journalists.^13^ An alternative perspective is that journalists cocreate narratives through which information is understood and circulated.^13^ Others involved in this cocreation include laypeople and professionals in, for example, media companies, health services, government, the pharmaceutical industry, charities and patient groups. Exploring the narrative structure of these health stories may help us to understand how they are received by readers and how they may influence decisions and behaviours around engagement with research. Previous work analysing narratives has focussed on specific health topics such as cancer^14^, ^15^, ^16^ or particular events such as the Northwick Park trial.^5^ The aim of this study was to explore how newspaper articles present stories about medical research and the ways in which people read them. We have used the example of RA research to explore this issue in depth.

## Methods

Our research comprised two separate elements, both using qualitative methods:

1. Analysis of newspaper articles relating to both RA and research.
2. Analysis of existing transcripts of interviews with patients with RA and their carers.

We used LexisNexis,^17^ a database of UK newspapers to search online and print versions of the 10 national newspaper groups with the highest circulation^1^ (see Table 1). For comparative purposes, we selected the cities of Newcastle upon Tyne and Birmingham to search all the local newspapers for these areas. Newcastle and Birmingham Universities have internationally renowned research groups studying RA; both areas have hospital trusts with large tertiary centres for rheumatology. Due to the short‐term influence of events, such as the Northwick Park trial or changes to legislation, on the coverage of research,^5^, ^16^ we searched articles appearing over a 10‐year period from 19 March 2004 to 18 March 2014.

**Table 1:** News sources

News source	Articles
The mail	240
The times	158
The telegraph	134
The express	123
The guardian	100
The mirror	65
The independent	59
The sun	32
The people (weekly title only)	2
The star	2
National total	915
Birmingham evening mail	24
Birmingham post	11
Sunday mercury	2
Birmingham total	37
Newcastle evening chronicle	37
Newcastle journal	35
Newcastle total	72

The number of newspaper articles from each news source (including daily and weekly titles) containing the search terms ‘rheumatoid arthritis’ and ‘research’ and with content relating to medical research for the 10‐year period from 19 March 2004 to 18 March 2014.

We identified articles containing both ‘rheumatoid arthritis’ and ‘research’. These search terms were chosen to capture articles about any type of research with relevance to RA. Where possible, we also searched the websites of the individual newspapers for the same time period using the search term ‘rheumatoid arthritis’. As the focus of the study was on representations of research, we excluded several kinds of article that were identified by these search terms, such as items about charity fund‐raising events or ‘research’ referring to information‐seeking behaviour.

In addition to the above, we also used an existing data set of transcripts from interviews with a total of 42 people, with 20 identified as containing talk about medical research in the news media. Each interviewee was either a person diagnosed with RA or a friend or family member with a physical and/or emotional role in the person's care. Participants were identified and approached at secondary and tertiary rheumatology clinics. Face to face, semi‐structured interviews were conducted, audio‐recorded and transcribed verbatim. These data were collected between 2012 and 2014 as part of a project exploring how people learn about RA, which included a focus on sources of information. All interviewees gave written informed consent prior to participation and the study was approved by NRES Committee North West – Cheshire (ref 12/NW/0148). We searched the transcripts for mention of ‘news’, ‘paper’, ‘article’, ‘report’ or ‘headline’ and began analysis work on the texts identified. Concurrently, we also reviewed all the transcripts to confirm that all relevant data had been captured in our initial search.

The analysis was conducted according to the standard procedures of rigorous qualitative analysis.^18^ Intellectually, we drew on ideas from the analysis of media in discourse analysis^19^ and ethnomethodology.^20^ In the early stages, we employed procedures from first‐generation grounded theory, open and focussed coding on subsets of data both independently and coming together as a group to discuss, challenge and modify codes.^21^ Later, we developed tables, or ‘frameworks’ to express our emerging concept, which we used to interrogate wider sections of the data, checking for ‘fit’ and searching for cases to challenge or modify our concept.^22^ We analysed data from the newspapers and interviews separately, but with each piece of work informing the other. Throughout the process, we undertook memoing^21^ (recording and developing analytic notes about ideas emerging from data analysis), mapping^23^ (creating visual representations of relationships among codes and categories) and deviant case analysis^24^ (sampling for and in depth study of anomalous cases).

## Results

### News sources

Our search yielded 1024 articles from all 10 of the national newspaper groups (see Table 1), after the removal of duplicates. The Mail group published most articles, amounting to 26% (240/915) of the national total. The same search identified articles from all five local newspapers in Newcastle and Birmingham (see Table 1).

### Article type or main focus

Table 2 shows the type or main focus of the articles in the data set. Our analysis work focussed on the *Trial of New Drugs or Medical Technologies* category as most relevant to medical researchers publicizing their work. The set of categories reflects all the contexts in which medical research was mentioned. The categories were generated through an iterative process of grouping and regrouping the articles. The categories appeared uniformly across the 10 year time period, with the exception of the *Northwick Park Trial* category, which was concentrated in a 2 year period following the event in 2006. The most common category was *Lifestyle and Alternative Medicine*. There were some differences in the distribution of articles between the local and national data. The national press published all the *Obituaries* and far more articles about *Lifestyle and Alternative Medicine* and *Business*. The local press published more articles relating to *Infrastructure*. There were no major differences between the Newcastle and Birmingham data sets.

**Table 2:** Article type or main focus

Category	Example	Newspaper articles			
		National	Newcastle	Birmingham	Total
Lifestyle and Alternative Medicine	Study showing taking cod liver oil reduced non‐steroidal anti‐inflammatory intake	282	12	8	302
Trial of New Drugs or Medical Technologies					
Focus RA	Trial of surgical implant for RA	80	15	5	100
Focus not RA	Trial of drug for psoriasis (which may also have potential in conditions such as RA)	88	2	6	96
Other Medical Research	Trial of nurse led clinics	102	10	2	114
Business	Company suffered losses after drug for RA disappointed in large‐scale studies	100	4	2	106
Northwick Park Trial	Victims may receive inadequate compensation	71	1	6	78
‘Research Shows’ (no information about study, only the findings)	Research shows that treating RA within 12 weeks gives the best chance of remission	63	4	2	69
Other	Local group hosting a talk on research in RA	54	9	2	65
DNA/Antibody/Biomarker	Discovery of test for an antibody predicting better response to a drug	47	3	0	50
Obituaries	Scientist who created transgenic mice, contributing to development of drugs for RA.	24	0	0	24
Infrastructure	New research centre and equipment funded via a grant.	4	12	4	20
Total		915	72	37	1024

The number of newspaper articles from each group of news sources (National, Newcastle upon Tyne, Birmingham) in each category.

### Positive and negative narratives

We carried out in depth analysis on the 100 articles in the *Trial of New Drugs or Medical Technologies, focus RA* category and identified two distinct narratives. The dominant narrative was a positive one, evident in 91 of 100 articles, while the second, less common narrative was a negative story of harm caused by clinical research. The negative narrative was apparent in only nine of the 100 articles and usually related to just two stories. The first story was the heart attack and death of a phase one trial volunteer in Edinburgh. The second story was the suspension of a repurposing trial of the drug Vioxx and the subsequent withdrawal of the drug. This negative narrative echoed reporting of the Northwick Park trial, previously analysed by Stobbart *et al*.^5^ Here, we treated the *Northwick Park Trial* as a separate category of 78 articles. Thus, to analyse this negative narrative, we reviewed the work of Stobbart *et al*.,^5^ the nine articles in the *Trial of New Drugs or Medical Technologies, focus RA* category and all the articles in the *Northwick Park Trial* category. Stobbart et al^5^ identified three themes: **human tragedy**;** good science and bad science**; and **engagement in science**. These themes were clearly evident in both categories of our data set. For example, an article in the Sun (Pete Woke With Foam Pouring Out His Mouth, 07 July 2008) characterized research staff as both attentive (**good science**) and predatory (**bad science**):

Bosses spotted an abnormality with his heart during a routine check and he was rushed to Edinburgh Royal Infirmary…

(attentive)

‘It's absolutely disgusting that these people can take advantage of someone in Peter's position’.

(predatory)

The positive narrative of medical research, present in 91 of 100 articles, was characterized by three essential components:

1. **Innovation** described the idea at the beginning of a study or the discovery at the end of a study. Novelty was stressed with words such as ‘breakthrough’, ‘revolutionary’, ‘pioneering’, ‘new approach’ and ‘world's first’.
2. **Hope** referred to an optimism that the innovation may eventually contribute to a reduction in the burden.
3. **Burden** was expressed as both personal suffering and societal cost. Emotive language was used to highlight the extreme nature of the personal burden, for example ‘crippling’, ‘debilitating’ and ‘constant agony’. Dramatic figures were employed to show the cost on a societal level, for example ‘700 000 sufferers’, ‘1000 a year have to give up work’ and ‘cost to the UK economy of the condition is at least £4.75 billion a year’.

In each article, these components were narratively related in the following format: *An **innovation** offers **hope** in a context of **burden.*** The following example from The Daily Telegraph (16 June 2008) was one of the shortest articles in the data set and as such contained the three essential components and little else:

Arthritis Hope
A new ‘smart’ drug (**innovation**) could save (**hope**) thousands of rheumatoid arthritis patients from years of pain and disability (**burden**)*,* research has shown. Tocilizumab, which is expected to be launched in Britain within six months, is being hailed as a major breakthrough (**innovation**).

### Characters and voices

There were recognizable patterns to the presentation of groups, such as patients and scientists, in the *Trial of New Drugs or Medical Technologies, focus RA* category and a key difference was the presence or absence of their views. **Characters** were described without any attributed quote or opinion, while **voices** were people, other than the journalist, giving a view. **Characters** appeared in descriptions of research activity, representing either the research team or the research subjects. For example:

**The Newcastle team** will test the jab in **eight people with the condition**.

(The Daily Mail, 14 August 2008)

**Characters** were identified solely in terms of role (e.g. ‘volunteers’), or with additional reference to location and/or institution (e.g. ‘scientists at Utah University in the United States’). Research team characters were always presented as active (e.g. ‘scientists found’; ‘researchers developed’). In contrast, research subject characters were usually presented as passive (e.g. ‘study using volunteers’; ‘patients are being given injections’). There were just two deviant cases to this passive subjects rule:

Patients **reported** reduced pain…

(The Daily Mail, 27 December 2011)

Patients **taking** the pill…

(The Express, 2 August 2012)

In contrast with the actions of the research team, even though the subject is presented as active, their action has no impact on others. Both articles also contain several examples of passive subjects.

**Voices** appeared with either a direct quote or an attributed belief or thought. **Voices** were either the research team or a commentator and the functions of each are described below. When a **voice** was a named individual, their credentials as a worthy spokesperson were confirmed in terms of their role and/or their institution. The following examples demonstrate the briefest and the most elaborate credentials from the data set, respectively:

GP Dr Ian Campbell…

(The Express, 2 August 2012)

Professor Gabriel Panayi, professor emeritus of rheumatology at King's College London and honorary consultant rheumatologist at Guy's and St Thomas…

(The Express, 8 February 2013)

**Voices** were sometimes groups rather than individuals and in such cases, there were no credentials (e.g. ‘doctors say’; ‘researchers hope’). There was a tendency for articles to mention an anonymous group first and later a named representative from that group.

Several functions were performed by the voices in articles: presenting the research; endorsing the research; and providing context. These functions overlapped, although **patient voices** never presented the research. The typical format was for the research team to present the research and a doctor or charity representative to endorse the research. Depending on the stage of the project, the team presented the research in terms of their aims or results, for example:

…says Dr Jane White head of clinical research at the company… ‘The first patients to have the implant, who were aged between 39 and 82, experienced a 60 per cent drop in pain levels’.

(The Daily Mail, 24 October 2006)

Commentators were not part of the research team and were usually a medical professional, academic and/or a representative of a charity (e.g. ‘Prof Alan Silman, medical director of Arthritis Research UK charity’). Patients were sometimes commentators, but only very rarely and in circumstances discussed below. The primary function of any commentator was to endorse the research.

**Figure 1:**
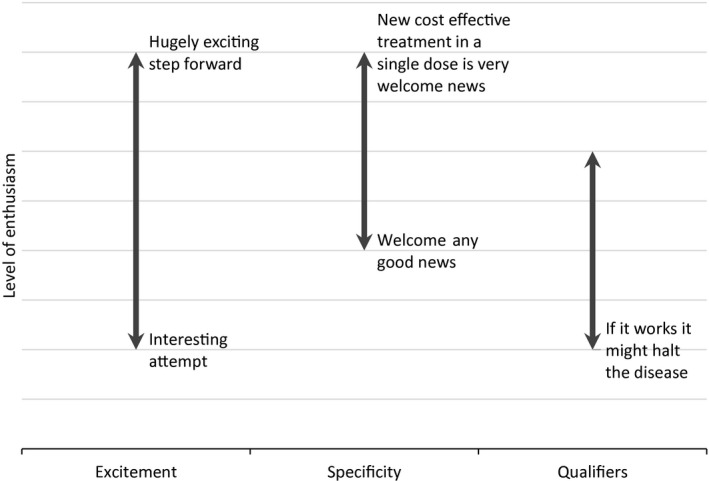
Continuum of enthusiasm. Factors contributing to the level of enthusiasm with which commentators endorse research.

Commentators endorsed research with varying levels of enthusiasm, indicated by a number of factors (see Fig. 1): firstly, the degree to which the research was described as exciting; secondly, the extent to which the research was welcomed in a general or a specific way; lastly, the presence or absence of qualifiers, such as ‘ifs’ and ‘buts’. The qualifications themselves clustered around just four categories: timing; uncertainty of outcome; limited application; and limited access to eventual treatment. The level of enthusiasm expressed did not appear to relate to the identity of the commentator or the stage of the research, with equally enthusiastic endorsements for the start of phase one and two trials as for the conclusion of phase three trials. The endorsements of Professor Alan Silman, the most frequent commentator in the data set, varied in enthusiasm. In essence, patients frequently featured in articles only as passive subjects with no attributed opinions. The views of the research team, other professionals and charity representatives were presented in strikingly uniform ways.

### Patient voices

**Patient voices** were relatively rare in the *Trial of New Drugs or Medical Technologies, focus RA* category, although more common in the Newcastle data: 7/80 National; 5/15 Newcastle; 0/5 Birmingham. When present, the key role of these **patient voices** was to contextualize the research. A **patient voice** was very often presented as a discrete entity, at the beginning or end of the article and sometimes as a separate piece with its own headline. **Patient voices** illustrated the **burden** of arthritis (an essential component of the positive medical research narrative described above) with descriptions of everyday struggles and losses. This served as a frame of reference for the presentation of **innovation** and **hope**. Furthermore, **patient voices** tended to end their stories with statements of **hope** as these final sentences show:

A lot of research is going on into the condition in the North East, which offers hope for sufferers.

(The Newcastle Journal 6 March 2012)

There were improvements straight away.

(The Daily Mail, 27 December 2011)

**Hope** was commonly associated with the research, but occasionally referred more generally to the impact of medications, as in the second example. In addition to contextualizing the research, some **patient voices** also endorsed the research. They did this with varying levels of enthusiasm, following the same pattern as for medical professionals, academics and charity representatives (see Fig. 1). Compared to other endorsers, patients tended to give endorsements with low specificity, for example:

I'm a great supporter of research into rheumatoid arthritis…

(The Newcastle Journal, 6 March 2012)

There was a standard format to the presentation of **patient voices**, which gave biographical information and, as already discussed, illustrated the **burden** of arthritis. We termed this the ‘minimum patient‐hood’ and found only two exceptions to the pattern. In the first instance the article was primarily about the patient's experiences of RA, with research a secondary focus. In the second instance, the patient was fulfilling two functions, both as a charity representative endorsing the research and as a patient illustrating the **burden**. The minimum patient‐hood comprised the following biographical information: first name; last name; age; location; occupation; and disease onset. This information suggested that the person was real and enabled the reader to imagine the person. Less information (e.g. omitting the last name or location) may have implied a pseudonym or made up example. Information about personal disease onset, established the authority of the **patient voice**. There were examples of **patient voices** presented with more than the minimum patient‐hood. The additional data comprised information about the person's family (e.g. ‘father of three’), age of disease onset (e.g. ‘first attack of rheumatoid arthritis came when I was aged 23’) and life before the disease (e.g. ‘was sports mad as a child’). These details were often present in longer articles and served to give a clearer, more relatable picture of the individual. Information about family may have been a proxy for occupation when a person was not in paid employment.

All 12 articles with a **patient voice** presented patients talking about their experience of the disease, but five of these patients made no mention of research involvement. In the remaining seven articles patients did mention research participation, usually highlighting positive outcomes, for example:

A glimmer of hope came when her consultant suggested she take part in a trial of an anti‐TNF drug. ‘It worked fantastically’.

(The Sun, 26 October 2006)

Only three patients talked about their day‐to‐day experience of participating in research, such as the following mention of attending hospital for regular infusions of medication:

I was eligible for RoActemra trials. I started the drug infusions in March. Every four weeks I go to the hospital and the drug is given via a drip. It takes a couple of hours but the results are impressive.

(Daily Mirror, 30 October 2009)

To summarize, there were few examples of **patient voices** in the *Trial of New Drugs or Medical Technologies, focus RA* category. **Patient voices** always contextualized the research by illustrating the **burden** of arthritis, but less commonly shared their practical experience of trial participation.

### Research into new treatments for other conditions

The *Trial of New Drugs or Medical Technologies, focus not RA* category, included treatments for a range of conditions including cancers, heart disease, Alzheimer's disease, multiple sclerosis and Crohn's disease. These studies were captured in our search because they either drew on knowledge from RA management or the work had potential future application for the treatment of RA. To understand whether our findings were transferable to other diseases, we tested our ideas against this data set. All our key findings were apparent in this category. There were far more positive than negative stories. All the positive stories included the narrative: *An **innovation** offers **hope** in the context of **burden***. Few of the articles had a **patient voice** and even fewer included patients’ experience of research involvement (see Appendix S1).

### How newspaper articles are read

We have shown how newspaper representations of research and trials tend to fit a common formula. However, how are these newspaper articles understood and used by the public? To explore this we analysed existing interview transcripts from patients with RA and their carers. Our sample comprised 20 individuals, 10 of whom had a diagnosis of RA and 10 were friends or family members with a physical and/or emotional role in the person's care. The relationship between the patient and carer was most commonly spouse, but sometimes child, partner, sibling, friend or child‐in‐law. Interviewees gave their age range, with these varying from 20s to 70s, but most commonly 60s. 12 of the 20 interviewees were female.

People with arthritis and their friends and relatives discovered print media resources either through actively looking for such material, just happening upon it, or being directed to it by someone else. These other people included carers, family and friends alongside broader social networks like friends of friends.

I get phone calls as soon as there's something in the paper. Because the people who know I've got it, they'll think I've missed it, and that will help me.

(RA patient, F, 70s)

They did, at times, ascribe others motives for directing them to or physically giving them articles. These ranged from demonstrating acts of care to, in one case, something like guilt, when another patient with well‐controlled RA contacted an unwell patient with news of an innovation, which may enable the unwell patient to also improve.

The findings suggest that people are not uncritical in their reading and can be cynical about how innovations are described by the press.

Massive headlines of the Daily Mail or the Daily Express, or something like that, for a miracle cure for rheumatoid arthritis … And you think ‘oh yeah, oh yeah, take that with a pinch of salt’.

(husband of RA patient, 60s)

All interviewees undertook work to contextualize the stories presented, such as asking those with relevant knowledge, say within the pharmaceutical industry, to translate the potential impact and timeline of an innovation. Thus, people can read articles with knowledge of the potential for newspaper articles to be partial or exaggerated.

The data show how people engage with and use the content of the articles in a variety of ways. Some read stories of innovation out of general interest in keeping updated, to see ‘if there's something recent’ (wife of RA patient, 60s). Some want to learn more about the personal stories of other people with RA or to generate a sense of hope for their own future. Others used the information within articles to assess their own care and treatments, for example noting that they have already ‘had that’ specific drug. They also reported discussing the stories in clinic, with some patients taking a copy with them, or in the case of a report on an on‐going trial, asking about potential eligibility, or even contacting the trial team directly. Alongside this, articles were discussed with or given to carers, to inform them, or they were ‘filed’, either literally or metaphorically.

## Discussion

We know that newspapers are read by a high percentage of the population^1^ and they are a source of information about health and research.^2^ Furthermore, it is likely that newspaper articles contribute to many people's perceptions of medical research, which in turn influence decisions and behaviours around engagement with research.^5^, ^6^ Our results show that newspaper articles of numerous different type and foci discuss research related to RA (see Table 2). 10% (100/1024) of articles relate specifically to research into new drugs and medical technologies to treat arthritis. This is a substantial amount given the breadth of articles reviewed. With the notable exception of the Northwick Park trial, the narrative is overwhelmingly positive and uniform with the same essential components and narrative presented in all positive stories: *An **innovation** offers **hope** in a context of **burden.***

There are well documented concerns about the accuracy and completeness of many reports.^7^, ^8^, ^9^, ^10^, ^11^, ^12^ None of these articles assess the ways these stories are read and they tend to assume a linear perspective and a passive readership. Kitzinger^25^ investigated audience understandings of AIDS reporting and found that even groups with no special knowledge of AIDS, presenting themselves as entirely dependent on the media for their information, were nevertheless drawing on other sources of knowledge such as friends, family and personal experience. These results coincide with contemporary theories of ‘active audiences’, reading with resistance as well as alignment to dominant ideas.^2^ Health information seeking, including from printed sources such as newspapers, is a component of health literacy – the capacity to obtain, process and understand basic health information and services to make appropriate health decisions.^26^ Recent work builds on the concept of health literacy as an individual risk or asset,^27^ describing ‘distributed health literacy’^28^ as a potential shared resource of individuals’ skills within social networks. Our analysis of interview transcripts suggests that patients and carers read newspaper articles critically and draw on other sources, such as friends or family with relevant knowledge, for interpretation and verification. These findings support the theories of both ‘active audiences’ and ‘distributed health literacy’. The potential for newspaper articles to misinform the public may be less of a concern than the cynicism with which some patients and carers viewed newspaper reports. This cynicism may be influenced by the striking uniformity of the narrative, as well as possible inaccurate or incomplete reporting. It is unclear what impact a cynical view of newspaper reporting of research might have on actual research engagement.

Understanding the essential components and narrative of positive stories about medical research (*An **innovation** offers **hope** in a context of **burden**)* may promote mass media publication by helping researchers to ‘package’ their research results in an appealing way for journalists. Conversely it may also enable researchers to challenge the status quo and argue a case for presenting stories about research which do not fit this narrative. Examples of this would be stories comprehensively addressing risks, uncertainty and timescales or stories highlighting patients’ experiences of participation. An awareness of the power of newspaper reports to impact upon behaviours, particularly to generate large volumes of participation enquiries,^5^, ^6^ should enable researchers to anticipate and make provision for this eventuality.

The rarity of patient stories and **patient voices** in newspaper articles about trials of new drugs or medical technologies is both surprising and notable. During the late 19^th^ and early 20^th^ centuries, there was a shift in newspaper content away from the simple reporting of macro events and towards the inclusion of personal stories to illustrate the impact of these events on the lives of ordinary people. This trend of personalization was described by Hughes^29^ in 1940 and has been explored more recently as one of many forms of popularization by which journalism engages audience attention.^13^ In contrast, our results suggest a ‘depersonalization bias’, conceptualized by Hallin and Briggs^13^ to explain journalists accepting linear views of the flow of health information and thus ‘privileging administrative perspectives at the expense of looking seriously at lay experience and understanding’. Kitzinger^30^ identified a similar privileging of official sources in media reporting of risk. There is evidence that patients seek out others’ stories related to health and illness to use as a resource, for example, when identifying options^31^ and making decisions.^32^ It is therefore likely that patients also look for stories about medical research involvement to explore options and make decisions about this. We found further evidence that patients look for personal stories in our interview transcript analysis.

We have explored the narratives present in the text of newspaper articles about medical research and the ways in which newspaper articles are read by patients with RA and their carers. Our analysis was developed using articles focussing on RA research and tested using articles about research in many other conditions. We identified the articles relating to other conditions from our original search for RA research. These articles all mentioned RA, which may have biased this sample towards immunological research. Our analysis of representations of medical research does not extend to the pictures in newspaper articles or to other sources of information such as television, radio or the internet. Thus, our conclusions cannot apply to these alternative sources. A focus on newspaper *representations* has been noted in many areas of media studies, with less known about the reception of newspaper articles.^2^

## Conclusion

Newspaper articles relating to research into new drugs and medical technologies are overwhelmingly positive and contain a simple narrative with three essential components: *An **innovation** offers **hope** in a context of **burden***. An awareness and understanding of this narrative may enable researchers to gain mass media exposure for their work and challenge this conventional style of reporting.

Our analysis of interview data on the interpretation and using of newspaper representations of medical research is a valuable contribution to a neglected area of study. Patients and carers read stories about medical research critically and sometimes with cynicism. This cynicism may be a consequence of the uniformity of the narrative, as well as possible inaccuracies or incompleteness. The effects of this cynicism on research engagement should be explored.

**Patient voices** and patient stories about research involvement were extremely rare in articles relating to research into new drugs and medical technologies. This finding conflicts with the historical trend in newspaper reporting towards more personal stories and the evidence suggesting that people would seek and use such stories. Further work is needed to explore the production of newspaper articles about medical research, which may shed light on the lack of patient stories and voices.

## Sources of funding

This work was funded by Arthritis Research UK, Chesterfield, UK (Project Grant Number 20018) and supported by the National Institute for Health Research, London, UK, through the Clinical Research Network. The work of Dr Paul Whybrow and Dr Richard Lee was funded by Arthritis Research UK, Chesterfield, UK (Project Grant Number 19624).

## Conflict of Interest

No conflict of interest declared.

## Supporting information


**Appendix S1:** Comparison of the subcategories *focus RA* and *focus not RA (Trial of New Drugs or Medical Technologies)*.
