# Supplementary material for: Drug breakthrough offers hope to arthritis sufferers: qualitative analysis of medical research in UK newspapers
Source: Health Expect. 2016 May 4;20(2):309–20. doi: 10.1111/hex.12460 (PMC5354054; doi:10.1111/hex.12460)
Supplement: Supplementary file 1 — Appendix S1: Comparison of the subcategories focus RA and focus not RA (Trial of New Drugs or Medical Technologies). [file HEX-20-309-s001.docx]

**Comparison of the sub-categories *focus RA* and *focus not RA (Trial of New Drugs or Medical Technologies)***

Negative or positive stories

|  | **Positive** | **Negative** | **Total** |
| --- | --- | --- | --- |
| **Focus RA** | 91 | 9 | 100 |
| **Focus Not RA** | 87 | 9 | 96 |

Presence or absence of patient voices in positive stories

|  | **No patient voice** | **Patient voice** | **Total** |
| --- | --- | --- | --- |
| **Focus RA** | 79 | 12 | 91 |
| **Focus Not RA** | 73 | 14 | 87 |

Content attributed to patient voice

|  | **Burden of disease only** | **Outcome of research participation** | **Day to day experience of research participation** | **Total Patient voices** |
| --- | --- | --- | --- | --- |
| **Focus RA** | 5 | 4 | 3 | 12 |
| **Focus Not RA** | 4 | 3 | 7 | 14 |

| **Other conditions targeted in the *focus not RA* sub-category** | |
| --- | --- |
| Alzheimer’s disease | Leukaemia |
| Asthma | Liver disease |
| Autoimmune diseases | Macular degeneration |
| Back pain | Miscarriage |
| Bipolar disorder | Motor neuron disease |
| Blindness | Multiple sclerosis |
| Blocked arteries | Obesity |
| Burns | Osteoarthritis |
| Cancer | Parkinson's disease |
| Cervical cancer | Premature birth |
| Coeliac disease | Psoriatic arthritis |
| Crohn’s disease | Psoriasis |
| Dementia | Prostate cancer |
| Depression | Sjorgren’s syndrome |
| Headache | Sleep disorders |
| Heart attack | Spinal cord injury |
| Heart disease | Stroke |
| Hepatitis | Tennis elbow |
| High blood pressure | Transplant |
| High cholesterol | Type 1 diabetes |
| HIV and AIDS | Type 2 diabetes |
| Infertility | Ulcerative colitis |
| Irritable bowel disease | Vision disorders |
| Juvenile idiopathic arthritis | Wounds |
| Leg ulcers |  |
